# Genetic Analyses Reveal a Role for Vitamin D Insufficiency in HCV-Associated Hepatocellular Carcinoma Development

**DOI:** 10.1371/journal.pone.0064053

**Published:** 2013-05-29

**Authors:** Christian M. Lange, Daiki Miki, Hidenori Ochi, Hans-Dieter Nischalke, Jörg Bojunga, Stéphanie Bibert, Kenichi Morikawa, Jérôme Gouttenoire, Andreas Cerny, Jean-François Dufour, Meri Gorgievski-Hrisoho, Markus H. Heim, Raffaele Malinverni, Beat Müllhaupt, Francesco Negro, David Semela, Zoltan Kutalik, Tobias Müller, Ulrich Spengler, Thomas Berg, Kazuaki Chayama, Darius Moradpour, Pierre-Yves Bochud, the Hiroshima Liver Study Group, the Swiss Hepatitis C Cohort Study Group

**Affiliations:** 1 Division of Gastroenterology and Hepatology, Centre Hospitalier Universitaire Vaudois, University of Lausanne, Lausanne, Switzerland; 2 Medizinische Klinik 1, Klinikum der J. W. Goethe-Universität Frankfurt a.M., Frankfurt a.M., Germany; 3 Laboratory for Digestive Diseases, Center for Genomic Medicine, RIKEN, Hiroshima, Japan; 4 Division of Frontier Medical Science, Programs for Biomedical Research, Department of Gastroenterology and Metabolism, Graduate School of Biomedical Sciences, Hiroshima University, Hiroshima, Japan; 5 Department of Internal Medicine I, University of Bonn, Bonn, Germany; 6 Service of Infectious Diseases, Department of Medicine, Centre Hospitalier Universitaire Vaudois, University of Lausanne, Lausanne, Switzerland; 7 Liver Unit, Ospedale Moncucco, Lugano, Switzerland; 8 University Clinic of Visceral Surgery and Medicine, Inselspital, Bern, Switzerland; 9 Institute for Infectious Diseases, University of Berne, Berne, Switzerland; 10 Division of Gastroenterology and Hepatology, University Hospital Basel, Basel, Switzerland; 11 Hôpital Neuchâtelois, Neuchâtel, Switzerland; 12 Division of Gastroenterology and Hepatology, University Hospital Zürich, Zürich, Switzerland; 13 Division of Gastroenterology and Hepatology, University Hospital Geneva, Geneva, Switzerland; 14 Division of Gastroenterology and Hepatology, Kantonsspital St. Gallen, St. Gallen, Switzerland; 15 Department of Medical Genetics, University of Lausanne, Lausanne, Lausanne, Switzerland; 16 Medizinische Klinik mit Schwerpunkt Hepatologie und Gastroenterologie, Charité Campus Virchow Klinikum, Berlin, Germany; 17 Sektion Hepatologie, Klinik und Poliklinik für Gastroenterologie und Rheumatologie, Universitätsklinikum Leipzig, Leipzig, Germany; University College London, United Kingdom

## Abstract

**Background:**

Vitamin D insufficiency has been associated with the occurrence of various types of cancer, but causal relationships remain elusive. We therefore aimed to determine the relationship between genetic determinants of vitamin D serum levels and the risk of developing hepatitis C virus (HCV)-related hepatocellular carcinoma (HCC).

**Methodology/Principal Findings:**

Associations between *CYP2R1*, *GC*, and *DHCR7* genotypes that are determinants of reduced 25-hydroxyvitamin D (25[OH]D_3_) serum levels and the risk of HCV-related HCC development were investigated for 1279 chronic hepatitis C patients with HCC and 4325 without HCC, respectively. The well-known associations between *CYP2R1* (rs1993116, rs10741657), *GC* (rs2282679), and *DHCR7* (rs7944926, rs12785878) genotypes and 25(OH)D_3_ serum levels were also apparent in patients with chronic hepatitis C. The same genotypes of these single nucleotide polymorphisms (SNPs) that are associated with reduced 25(OH)D_3_ serum levels were found to be associated with HCV-related HCC (*P* = 0.07 [OR = 1.13, 95% CI = 0.99–1.28] for *CYP2R1*, *P* = 0.007 [OR = 1.56, 95% CI = 1.12–2.15] for *GC*, *P* = 0.003 [OR = 1.42, 95% CI = 1.13–1.78] for *DHCR7*; ORs for risk genotypes). In contrast, no association between these genetic variations and liver fibrosis progression rate (*P*>0.2 for each SNP) or outcome of standard therapy with pegylated interferon-α and ribavirin (*P*>0.2 for each SNP) was observed, suggesting a specific influence of the genetic determinants of 25(OH)D_3_ serum levels on hepatocarcinogenesis.

**Conclusions/Significance:**

Our data suggest a relatively weak but functionally relevant role for vitamin D in the prevention of HCV-related hepatocarcinogenesis.

## Introduction

Chronic hepatitis C is associated with important morbidity, resulting in liver cirrhosis and its complications in a significant proportion of infected individuals [1]. Due to the rising age of the hepatitis C virus (HCV)-infected population in the Western world, a dramatic increase of cases with advanced liver cirrhosis and hepatocellular carcinoma (HCC) has been predicted for the next decade [1]. Due to the insufficient treatment options for advanced HCC as well as limited number of liver allograft donors for patients with curable disease, improved strategies to screen for early HCC or to prevent the development of HCC are warranted [2]. Recently, two genome-wide association studies (GWAS) have identified single nucleotide polymorphisms (SNPs) in the region of *DEPDC5* and *MICA* as susceptibility loci for HCC development in patients with chronic hepatitis C [3], [4]. Although these genetic variations were strongly associated with HCV-induced HCC, with potentially important implications for the identification of patients at high risk of developing HCC, it might be challenging to translate these findings into novel therapeutic strategies for HCC.

Vitamin D insufficiency is common in different populations worldwide and has been associated with the presence of various types of cancer, including HCC [5]–[7]. These observations attracted considerable interest because vitamin D can be easily supplemented, with only infrequent side-effects and little costs. Yet, it has remained mostly unclear whether there is a causal association between vitamin D insufficiency and cancer development, or whether reduced vitamin D serum levels are simply surrogates for other circumstances in cancer patients (e.g. malnutrition, limited exposure to sunlight) [5]–[7]. Recently, two large GWAS have identified SNPs at three loci (*GC*, encoding the vitamin D binding protein; *DHCR7*, encoding 7-dehydrocholesterol reductase; and *CYP2R1,* encoding a liver 25-hydroxylase) as genetic determinants of reduced 25(OH)D_3_ serum levels [8], [9]. We hypothesized, that associations between these genetic variations and the occurrence of malignant or other diseases may provide a stronger argument for a causal relationship between vitamin D and the development of such diseases than the investigation of (punctual) vitamin D serum levels. In view of the high prevalence of severe vitamin D deficiency in patients with chronic hepatitis C [10], we therefore sought to investigate the association between genetic variations in *CYP2R1*, *GC*, and *DHCR7* and HCV-induced HCC.

## Methods

### Objectives

Genetic variations in three independent genes, *CYP2R1*, *GC*, and *DHCR7,* are associated with life-long reduced 25(OH)D_3_ serum levels [9]. Vitamin D insufficiency has been associated with the occurrence of various types of cancer, but causal relationships remain elusive. Therefore, we hypothesized that genetic determinants of 25(OH)D_3_ serum levels may be associated with HCV-related HCC if there is a causal relationship between vitamin D metabolism and HCC development in chronic hepatitis C patients. Hence, we assessed associations between the presence of HCC in HCV infected patients and genetic variants in *CYP2R1*, *GC*, and *DHCR7* in the primary analysis of the present study.

### Participants

For the primary analysis of the present study, the association between HCV-induced HCC and genetic variants in *CYP2R1*, *GC*, and *DHCR7*, four independent cohorts of HCV-infected individuals were investigated, two cohorts including Caucasians and two cohorts including Japanese individuals. All cohorts include consecutive patients from various outpatient clinics; hence the prevalence of HCC in our cohorts is not representative of the prevalence of HCC in HCV-infected patients in general.

The first Caucasian cohort was selected from patients enrolled in the Swiss Hepatitis C Cohort Study (SCCS). The SCCS is a multicenter study pursued at 8 major Swiss hospitals and their local affiliated centers, including a total of 3,648 patients with chronic or resolved HCV infection [11]. For the present analysis, SCCS patients were included in our study if they had chronic hepatitis C, had provided written informed consent for genetic testing, had genomic DNA available for testing, if they were Caucasian, and if the duration of infection with HCV was known. Patients with hepatitis B virus infection were excluded from the discovery cohort, as well as from the replication cohorts. A second, independent cohort of Caucasian patients with chronic HCV infection with or without HCC was identified (designated as Berlin/Bonn cohort in the following); these patients were recruited at the University Hospital Departments of Gastroenterology and Hepatology in Bonn, Berlin and Leipzig in Germany, as described in Nischalke *et al* [*12*]. In addition, two independent cohorts of Japanese patients with chronic hepatitis C with or without HCC were included (a detailed description of these cohorts is provided in Miki *et al.* [*4*]; the cohorts are designated as Japanese GWAS and Japanese Replication cohort, as in Miki *et al.*). Importantly, the duration of infection with HCV was known in patients of the SCCS but not in the three additional cohorts.

In addition to the primary analysis of this study, a number of sub-analyses were performed in order to validate our research strategy. These sub-analyses investigated possible associations between genetic variations in *CYP2R1*, *GC* and *DHCR7* and i) liver fibrosis progression rate (FPR), ii) response to treatment with pegylated interferon-α (PEG-IFN-α) and ribavirin, and iii) 25(OH)D_3_ serum levels. These sub-analyses were performed in SCCS patients only because of the thorough documentation of these end-points together with the known duration of infection in the SCCS. FPR was defined as a dichotomized phenotype (< *vs*. ≥ sex-adjusted median FPR), which was calculated on the basis of the ratio of the METAVIR fibrosis score to the estimated duration of infection in years until liver biopsy (METAVIR units per year), as described previously [13]. Hence, all SCCS patients with at least one available liver biopsy with fibrosis staging prior to antiviral treatment and with known date of infection were included in the analyses of FPR. The treatment response analyses was restricted to SCCS patients who were treated under clinical practice conditions with either PEG-IFN-α2a or PEG-IFN-α2b in combination with weight-based ribavirin, with standard treatment durations (48 weeks for HCV genotype 1 and 4, 24 weeks for HCV genotype 2 and 3), and if they had received ≥80% of the recommended dose of both agents during the first 12 weeks of therapy. SVR was defined as HCV RNA below the limit of detection in a sensitive assay ≥24 weeks after treatment completion, and all patients who failed to achieve SVR were classified as nonresponders. Serum concentrations of 25(OH)D_3_ were determined in all SCCS patients with chronic hepatitis C in whom a plasma sample at baseline of antiviral therapy or at the time of a liver biopsy was available. Demographic and clinical characteristics were extracted from clinical databases. High alcohol intake was defined as consumption >40 g per day over a period of ≥5 years. Liver biopsies were evaluated by experienced local pathologists. Liver fibrosis was classified according to the METAVIR score. Necroinflammatory activity was stratified into two groups, absent to mild activity *vs*. moderate to high activity. Steatosis was classified as absent or present. HCC was diagnosed either by biopsy or, in selected cases, by typical presentation in two independent imaging modalities. The study was approved by local ethical committees.

### Description of Investigations Undertaken

In a recent GWAS, Wang *et al.* have identified three loci (*CYP2R1, GC*, and *DHCR7*) to be associated with reduced 25(OH)D_3_ serum levels [9]. Importantly, a second independent GWAS by Ahn *et al.* yielded similar results and confirmed the association between the three loci and 25(OH)D_3_ serum levels [8]. From these studies, we selected the most significant SNPs for each locus (rs1993116 and rs10741657 in *CYP2R1*; rs2282679 in *GC*; rs7944926 and rs12785878 in *DHCR7*). rs10741657 and rs1993116 in *CYP2R1* as well as rs7944926 and rs12785878 in *DHCR7* are in high LD (Table S1). Hence, these SNPs can be substituted by each other for an investigation of the association between the indicated loci and HCV-related HCC, and either rs7944926 or rs12785878 in *DHCR7* and either rs10741657 or rs1993116 in *CYP2R1* were genotyped in each of the four cohorts. Specific SNPs were selected according to availability in our databases, as genotyping of these SNPs was either performed in the context of previous GWA studies of our cohorts [4], [14], or by using a fluorescent-based competitive allele-specific PCR genotyping system (KBioscience, UK) using the primers listed in Table S2. SNPs in *CYP27B1*, which we have previously shown to be associated with SVR [10], [15], were not included in the present study as they are believed to be predominantly important during the rapid regulation of calcitriol tissue levels in inflammatory responses, and because they have no impact on 25(OH)D_3_ serum levels.

Measurement of 25(OH)D_3_ was performed as described previously [10].

### Ethics

The study was approved by local ethical committees of each affiliation (Switzerland: Universitätsspital Basel, Basel; Inselspital, Bern; University Hospital Geneva, Geneva; CHUV, Lausanne; Ospedale Moncucco, Lugano; Hôpital Neuchâtelois, Neuchatel; Kantonsspital St. Gallen, St. Gallen; Universitätsspital Zürich, Zürich; Germany: University of Berlin, Berlin; University of Bonn, Bonn; Japan: University of Hiroshima, Hiroshima), and written informed consent was received from all participants.

### Statistical Analyses

Testing for Hardy-Weinberg equilibrium and linkage disequilibrium (LD) was performed with the genhw and pwld packages in Stata (version 9.1, StataCorp, College Station, TX). Associations of SNPs with risk of HCV-induced HCC (dichotomic variable HCC *vs.* no HCC) were assessed with χ^2^ contingency tables, and – in the SCCS in patients with known duration of infection – in uni- and multivariate Cox regression models. Associations between SNPs and treatment outcome (SVR *vs.* no SVR) and with FPR (below *vs*. above sex-adjusted median FPR) were assessed in logistic regression models and Cox regression models, respectively. In regression models, SNPs were analyzed using an additive model (none, one or two copies of the minor allele were coded 0, 1 and 2, respectively, assuming greater effect with increased copy number of the minor allele), unless otherwise specified.

## Results

### Patient Characteristics

Out of a total of 3,648 patients enrolled in the SCCS, 1661 patients with known duration of infection were included in the primary analyses of the present study based on the selection criteria defined above. Out of these patients, 50 individuals developed HCC. The three additional cohorts included a total number of 1229 patients with chronic hepatitis C and HCC, as well as 2714 patients with chronic hepatitis C without HCC at the time of the analysis. Thus, the primary analysis of the present study included 1279 chronic hepatitis C patients with HCC and 4325 without HCC. Baseline characteristics of these patients are summarized in Table 1.

**Table 1:** Baseline characteristics of included patients.

	SCCS		Japanese GWAS		Japanese Repl.		Bonn/Berlin	
	HCC	Control	HCC	Control	HCC	Control	HCC	Control
N	50	1611	310	1252	803	1253	116	209
Age (SD)^1^	28* (15)	21 (10)	–	–	–	–	61*(10.6)	48(12.4)
Male sex, n (%)^1^	31 (62)	991(62)	230 (74)*	725 (58)	515 (64)*	570 (45)	73 (58)	114 (55)
Alcohol (≥40 g/d ≥5 years), n (%)^2^	3 (8)	220 (17)	–	–	–	–	–	–
Diabetes, n (%)	9 (18)*	91 (6)	–	–	254 (32)*	219 (18)	–	–
HCV Genotype, n (%)^3^								
1, 4	27 (60)	1014 (64)	307 (99)	1241 (99)	540 (72)*	837 (67)	–	–
2, 3	17 (40)	570 (36)	2 (1)	10 (1)	213 (28)*	416 (33)	–	–

* These comparisons between HCC cases and controls are statistically significant (*P*<0.05). Repl, replication. The cohort labeling “Japanese GWAS” and “Japanese Replication” is based on the initial description of the cohorts in Miki *et al.*, for the present study both cohorts served as replication cohorts.

1 Age and sex data was missing in 5 patients in the Bonn/Berlin cohort. For the SCCS, age of infection is shown. Age of infection was unknown for the Bonn/Berlin cohort, for this cohort age at diagnosis is shown.

2 Alcohol consumption data was missing in 12 HCC and 290 non-HCC patients from the SCCS.

3 HCV genotype was missing in 5 HCC and 23 non-HCC patients from the SCCS, in 1 HCC and 2 non-HCC patients from the Japanese GWAS, and 50 HCC patients from the Japanese replication cohorts.

In addition, 963 and 750 SCCS patients were eligible to assess the impact of genetic variations in *CYP2R1*, *GC*, and *DHCR7* on FPR and on the outcome of standard treatment with PEG-IFN-α and ribavirin, respectively. Serum levels of 25(OH)D_3_ were only available in a minority for 496 SCCS patients.

### Association between Genetic Determinants of 25(OH)D_3_ Serum Levels and HCV-induced HCC

Median 25(OH)D_3_ serum levels in patients with chronic hepatitis C with or without HCC in the SCCS were 12.7 and 14.3 ng/mL, (range 4.9–40.9 and 3.9–76.9) respectively (*P* = 0.19, values available in 496 patients). However, 25(OH)D_3_ serum levels fluctuate strongly during seasons and as a consequence of numerous circumstances such as exposure to sunlight, nutrition, accompanying diseases, vitamin D supplementation and others. We therefore believe that genetic variants in *CYP2R1*, *GC*, and *DHCR7*, with their proven impact on 25(OH)D_3_ serum levels, should be used as surrogates for long-term 25(OH)D_3_ serum levels. Hence, we genotyped the most relevant tagging SNPs for these loci (rs1993116/rs10741657 for *CYP2R1*, rs2282679 for *GC*, rs7944926/rs12785878 for *DHCR7*; data on LD and Hardy Weinberg equilibrium for these SNPs are shown in Table S1), and assessed their association with HCV-related HCC.

Table 2 summarizes results of the primary analysis for associations between HCV-related HCC and genetic variations in *CYP2R1*, *GC,* and *DHCR7* in the four independent patient cohorts. In the combined analyses of these cohorts, the strongest association with HCV-induced HCC was found for *GC* (*P* = 0.007, OR = 1.56, 95% CI = 1.12–2.15) and *DHCR7* (*P* = 0.003, OR = 1.42, 95% CI = 1.13–1.78), whereas *CYP2R1* was almost significantly associated with HCV-induced HCC (*P* = 0.07, OR = 1.13, 95% CI = 0.99–1.28). Remarkably, in each independent cohort, frequencies and ORs for the risk alleles at each locus showed an association in the similar direction, even though statistical significance was not reached for all subgroups.

**Table 2:** Summary of associations between SNPs in *CYP2R1*, *GC*, and *DHCR7*, and HCV-related hepatocellular carcinoma development.

CYP2R1												
			Cases			Controls			Risk allele frequencies			
SNP	Study	Allele 1/2	11	12	22	11	12	22	Case	Control	*P*	OR (95% CI)
rs1993116	SCCS	A/G	6	16	28	199	774	634	0.72	0.64	0.02	1.95 (1.18–3.41)
rs1993116	JapaneseGWAS	A/G	41	136	133	163	621	468	0.65	0.62	0.07	1.26 (0.98–1.61)
rs10741657^1^	Japanese Replication	A/G	106	377	320	174	597	482	0.63	0.62	0.5	1.06 (0.88–1.27)
rs1993116	Bonn-Berlin	A/G	17	48	47	25	98	81	0.63	0.64	0.7	1.10 (0.67–1.76)
	***Combined***	A/G	***170***	***577***	***528***	***561***	***2090***	***1665***	***0.64***	***0.63***	***0.07***	***1.13 (0.99–1.28)***

*GC*												
SNP	Study	Allele 1/2	11	12	22	11	12	22	Case	Control	P	OR (95% CI)
rs2282679	SCCS	T/G	26	20	4	855	641	104	0.28	0.27	0.6	1.25 (0.44–3.54)
rs2282679	JapaneseGWAS*	T/G	153	125	31	679	475	97	0.30	0.27	0.19	1.33 (0.87–2.03)
rs2282679	Bonn-Berlin	T/G	55	46	15	117	77	14	0.33	0.25	0.06	2.01 (0.97–4.38)
	***Combined***	***T/G***	***234***	***191***	***50***	***1651***	***1193***	***215***	***0.31***	***0.27***	***0.007***	***1.56 (1.12–2.15)***

*DHCR7*												
SNP	Study	Allele 1/2	11	12	22	11	12	22	Case	Control	P	OR (95% CI)
rs7944926	SCCS	T/C	17	10	4	462	310	55	0.29	0.25	0.9	1.04 (0.51–2.14)
rs7944926	JapaneseGWAS	T/C	127	156	27	599	534	119	0.34	0.31	0.03	1.32 (1.03–1.70)
rs12785878^2^	Japanese Replication	T/G	84	336	383	153	543	557	0.69	0.66	0.15	1.13 (0.95–1.36)
rs12785878^2^	Bonn-Berlin	T/G	63	44	9	113	77	18	0.27	0.27	1.00	1.00 (0.63–1.58)
	***Combined*** #	***T/C***	***144***	***166***	***31***	***1061***	***844***	***174***	***0.33***	***0.29***	***0.003***	***1.42 (1.13–1.78)***

Allele 2 indicates the risk allele, according to Wang et al. [9]. *P*-values and ORs were calculated for risk genotypes using favorable genotypes as a reference, i.e. for *CYP2R1* by comparing GG *vs.* GA/AA genotypes, for *GC* by comparing TT/TG vs. GG genotypes, and for *DHCR7* by comparing TT vs. TC/CC genotypes.

# Only patients from the SCCS and Japanese GWAS were included in the combined analysis for this locus, because of the different allele frequencies for rs12785878 in Japanese patients compared to Caucasian patients. Data remain significant after inclusion of the Bonn-Berlin cohort (P = 0.018, OR = 1.27 [95% CI = 1.04–1.56]). *Genotyping of this SNP failed in the Japanese Replication cohort due to limited amounts of DNA. Please note that the total number of patients with available genotypes is not equal between different loci due to limited amount of DNA or genotyping failure in some cases.

1 rs1993116 and rs10741657 are in complete LD in the Caucasian and Japanese population (R^2^ = 0.95 and = 1.00, respectively), the major alleles of both SNPs have a similar impact on 25(OH)D_3_ serum levels, indicating that both SNPs can be used equivalently [9].

2 rs7944926 and rs12785878 are in complete LD in the Caucasian and Japanese population (R^2^ = 1.00 and = 1.00, respectively), the major alleles of both SNPs have a similar impact on 25(OH)D_3_ serum levels, indicating that both SNPs can be used equivalently [9].

Of note, genotyping of rs1287578 in *DHCR7* revealed huge differences of T allele frequencies between Caucasian and Japanese cohorts. These differences are in line with allele frequencies reported in HapMap. Since previous GWAS on vitamin D serum levels did not include relevant numbers of Asian individuals, a functional interpretation of rs1287578 genotyping data in Japanese appears to be difficult, as the risk allele for this SNP in Asians has not yet been clearly identified. Therefore, data for rs1287578 genotype in the Japanese replication cohort were not included in the combined analysis shown in Table 2.

**Figure 1:**
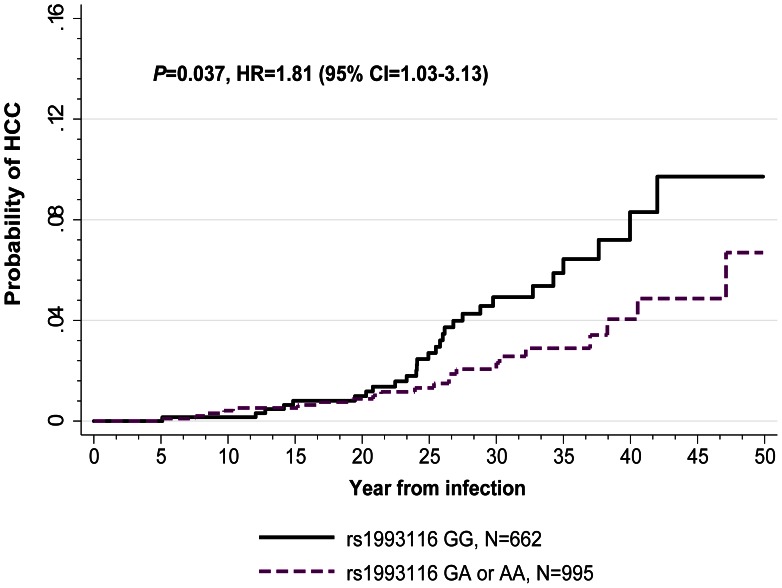
Risk of hepatocellular carcinoma (HCC) development in SCCS patients with chronic hepatitis C and known duration of infection, according to *CYP2R1* rs1993116 genotypes. The probability to develop HCC from the time of hepatitis C virus infection by *CYP2R1* rs1993116 genotypes (GG *vs*. GA/AA) was assessed by using cumulative incidence curves, with censoring of data at the date of last follow-up or death. Statistics are shown for univariate Cox regression analysis. CI, confidence interval; HR, hazard ratio.

The known duration of infection allowed an additional cox regression analyses of the risk of HCC in patients from the SCCS. Figure 1 shows the cumulative incidence of HCC in patients with the *CYP2R1* risk genotype rs1993116 GG compared to those with the favorable genotypes GA and AA (*P* = 0.037, hazard ratio (HR) = 1.81 (95% confidence interval (CI) = 1.03–3.13).

To exclude a possible selection bias within the SCCS, a subanalysis was performed in which the inclusion criterion “known duration of infection”, which was specific for the SCCS, was omitted. As shown in Table S3, results of this case-control study are largely comparable to the primary analysis of our study.

### Association between Genetic Determinants of 25(OH)D_3_ Serum Levels and Liver Fibrosis Progression Rate (FPR) and Treatment Outcome

Thus far, we cannot completely exclude that the above described associations between genetic determinants of reduced 25(OH)D_3_ serum levels and HCV-induced HCC are primarily mediated by an effect of the indicated SNPs on FPR or treatment outcome. We therefore performed sub-analyses to test whether FPR or treatment outcome are associated with variations in *CYP2R1*, *GC* and *DCHR7*. In 963 SCCS patients in whom FPR could be calculated, none of the SNPs was significantly associated with slow *vs*. fast FPR (*P* = 0.2 for rs1993116 in *CYP2R1*; *P* = 0.5 for rs2282679 in *GC*, and *P* = 0.3 for rs7944926 in *DHCR7*; Table 3). In addition, in 750 SCCS patients who had received standard therapy with PEG-IFN-α and ribavirin, no significant associations were found between SNPs in *CYP2R1*, *GC* and *DHCR7* and treatment outcome (SVR *vs.* no SVR; *P* = 0.9, 0.4, 0.2, respectively; Table 4), suggesting that the observed associations between these loci and HCC are specific for (HCV-induced) hepatocarcinogenesis. Finally, we calculated 25(OH)D_3_ serum levels according to *CYP2R1*, *GC*, and *DHCR7* genotypes. 25(OH)D_3_ serum levels were 14.9, 13.4 and 12.4 ng/mL in patients with *CYP2R1* rs1993116 genotype AA, AG, and GG (*P* = 0.41), respectively; 14.4, 14.2, 11.7 in patients with *GC* rs2282679 genotype TT, TG, GG (*P* = 0.037); and 14.3, 14.4, and 13.1 in patients with *DHCR7* rs7944926 genotype TT, TC, and CC (*P* = 0.44).

**Table 3:** Association of SNPs in *CYP2R1*, *GC*, and *DHCR7* with liver fibrosis progression rate (FPR) in patients with chronic hepatitis C.

			Patients, n (%)			
Gene	SNP	Gt	Slow FPR*	Fast FPR*	P	OR (95% CI)
***CYP2R1***	rs1993116	GG	183 (41)	192 (37)		
		GA	213 (48)	263 (51)	0.2	1.20 (0.93–1.56)
		AA	47 (11)	65 (13)		
***GC***	rs2282679	TT	236 (53)	266 (51)		
		TG	175 (40)	217 (42)	0.5	1.08 (0.84–1.39)
		GG	31 (7)	34 (7)		
***DHCR7***	rs7944926	TT	160 (54)	221 (58)		
		TC	115 (39)	139 (36)	0.3	0.85 (0.63–1.16)
		CC	22 (7)	22 (6)		

* FPR, liver fibrosis progression rate. Slow *vs.* fast FPR was defined as FPR<vs. ≥ sex-adjusted median FPR in METAVIR-units per year. Statistics are shown for logistic regression analyses based on the additive model of inheritance. The usage of other models of inheritance (recessive, dominant) did not result in significant associations as well (not shown).

**Table 4:** Association of SNPs in *CYP2R1*, *GC*, and *DHCR7* with response to treatment of pegylated interferon-α and ribavirin in patients with chronic hepatitis C.

			Patients, n (%)			
Gene	SNP	Gt	without SVR	with SVR	P	OR (95% CI)
***CYP2R1***	rs1993116	GG	109 (38)	171 (37)		
		GA	138 (48)	238 (52)	0.9	1.03 (0.76–1.39)
		AA	42 (15)	52 (11)		
***GC***	rs2282679	TT	166 (57)	248 (54)		
		TG	105 (36)	186 (40)	0.42	1.14 (0.85–1.54)
		GG	19 (7)	26 (6)		
***DHCR7***	rs7944926	TT	127 (53)	230 (58)		
		TC	95 (40)	143 (36)	0.2	0.81 (0.59–1.12)
		CC	17 (7)	22 (6)		

Statistics are shown for logistic regression analyses based on the additive model of inheritance. The usage of other models of inheritance (recessive, dominant) did not result in significant associations as well (not shown).

## Discussion

The present study shows that genetic variations in *CYP2R1, GC*, and *DHCR7* are associated with progression to HCC in patients with chronic hepatitis C. The SNPs investigated in *CYP2R1, GC*, and *DHCR7* have been identified by two independent GWAS as relevant genetic determinants of reduced 25(OH)D_3_ serum levels [8], [9]. Although genetic association studies cannot prove causal relationships between genetic variations and specific phenotypes, the association here identified between three genetically independent, but functionally related susceptibility loci of vitamin D deficiency and HCV-induced HCC suggest that an impaired vitamin D metabolism (functionally) contributes to hepatocarcinogenesis in HCV-infected patients. Importantly, the lack of association of these SNPs in *CYP2R1, GC*, and *DHCR7* with FPR and response to treatment with PEG-IFN-α and ribavirin suggests a specific effect of these genetic variations on HCV-induced hepatocarcinogenesis.

Vitamin D insufficiency has been previously linked to the development of HCC [16]. However, causal relationships remained mostly unclear because these studies were small or concentrated on the assessment of 25(OH)D_3_ serum levels at the date of HCC, which may result in false-positive associations due the influence of impaired liver function on circulating 25(OH)D_3_ [5], [16]–[18]. In addition, randomized controlled clinical trials evaluating the effect of vitamin D or its analogues on HCC development are lacking. Thus, the results of the present, large-scale genetic association study add a significant argument to evaluate the possible chemo-preventive effect of vitamin D supplementation in HCV-infected patients. However, although our data suggest a causal role of vitamin D metabolism in HCV-induced HCC, there is currently no published evidence that vitamin D supplementation would translate into a benefit in HCV-infected patients with advanced liver disease. In this regard, caution is advisable since some placebo-controlled studies evaluating the supplementation of other vitamins have reported a reduced overall survival rate in some verum groups [19]. Yet, in view of our present data, and in view of the high prevalence of severe vitamin D deficiency in patients with chronic hepatitis C [10], [20], [21], randomized controlled clinical trials in HCV-infected patients with advanced liver disease appear to be justified.

### Limitations

Our study has potential limitations. First, 25(OH)D_3_ serum levels were only available in a relatively small subgroup of patients. Nevertheless, we observe the same trends for 25(OH)D_3_ serum levels according to *CYP2R1, GC*, and *DHCR7* genotypes as described previously, and HCV-infected patients with HCC had slightly lower 25(OH)D_3_ serum levels compared to those without HCC. More important, we believe that analyses of associations between punctual 25(OH)D_3_ serum levels and endpoints such as HCC can be misleading, since 25(OH)D_3_ serum levels strongly fluctuate during seasons, with age, and as a consequence of numerous other conditions (liver fibrosis, diabetes, obesity, supplementation, etc.) [6], [7]. A second limitation of our study is the relatively weak level of statistical significance in the analyses of the individual cohorts, with the consequence that associations for the three loci with HCC became only fully significant in the combined analyses of all included patients. Most likely, this can be explained by the relatively low frequency of the risk genotypes (especially of *GC* and *DHCR7*), as well as by the numerous variables with influence 25(OH)D_3_ serum levels in a given individual [9]. In this regard, it is important to note that ORs for the risk genotype of all genes (*CYP2R1, GC*, and *DHCR7*) were similarly directed in the discovery cohort as well as in all replication cohorts, confirming a true association between these loci and HCV-induced HCC. In addition, the strengths of the associations between these loci and HCV-induced HCC were largely comparable to the strength of the associations between these loci and 25(OH)D_3_ serum levels (strong effect of *GC* and *DHCR7*, moderate effect of *CYP2R1*, according to Wang *et al*.) [9]. Nevertheless, the observed subtle differences between the different cohorts included in our study may not only be explained by the relatively small sample size of individual cohorts, but also by specific cohort features such as different recruitment strategies (e.g. cohort study *versus* case-control studies). In this regard, our study also cannot fully rule out whether all investigated genes (*CYP2R1, GC, DHCR7*) have the same impact on progression to HCV-related HCC at different stages of liver disease or in populations of different ancestries. Furthermore, our study cannot clearly characterize whether the observed findings apply for patients who did or did not respond to antiviral therapy. Though we show in the SCCS (in a minority of the whole study population), that SNPs in *CYP2R1*, *GC*, and *DHCR7* were not associated with treatment outcome, it remains unclear whether these genetic variations are associated with HCC in both individuals with or without treatment-induced eradication of HCV.

The heterogeneity of the four independent cohorts included in our analyses might be perceived as another limitation. This applies for important cohort features such as race, recording of treatment modalities, HCC frequency, or different allele frequencies for some SNPs (especially rs1278578).

### Conclusions

In conclusion, we provide evidence for a functionally relevant contribution of reduced 25(OH)D_3_ serum levels to HCV-induced HCC. Controlled clinical trials to evaluate the impact of vitamin D supplementation on HCC risk and overall survival in patients with chronic hepatitis C appear to be justified.

## Supporting Information

Table S1 **Linkage disequilibrium of SNPs in** ***CYP2R1*** **,** ***GC*** **, and** ***DHCR7*** **investigated in the present study.**
(DOC)

Table S2 **Primers for SNP genotyping assays.**
(DOC)

Table S3 **Summary of associations between SNPs in** ***CYP2R1*** **,** ***GC*** **, and** ***DHCR7*** **, and HCV-related hepatocellular carcinoma development, considering the SCCS as case-control study.**
(DOC)
