# Supplementary material for: Genetic Analyses Reveal a Role for Vitamin D Insufficiency in HCV-Associated Hepatocellular Carcinoma Development
Source: PLoS One. 2013 May 29;8(5):e64053. doi: 10.1371/journal.pone.0064053 (PMC3667029; doi:10.1371/journal.pone.0064053)
Supplement: Table S1 — Linkage disequilibrium of SNPs in CYP2R1 , GC , and DHCR7 investigated in the present study. (DOC) [file pone.0064053.s001.doc]

**Table S1. Linkage disequilibrium of SNPs in *CYP2R1*, *GC*, and *DHCR7* investigated in the present study.**

| Gene | SNP | rs1993116 | rs2282679 | rs7944926 | rs10741657 | rs12785878 |
| --- | --- | --- | --- | --- | --- | --- |
| *CYP2R1* | rs1993116 | 0.37 |  |  |  |  |
| *GC* | rs2282679 | 0.00 / 0.01 | 0.27 |  |  |  |
| *DHCR7* | rs7944926 | 0.00 / 0.04 | 0.00 / 0.02 | 0.26 |  |  |
| *CYP2R1* | rs10741657 | 0.95 / 0.99 | 0.00 / 0.04 | 0.00 / 0.04 | 0.36 |  |
| *DHCR7* | rs12785878 | 0.00 / 0.03 | 0.00 / 0.01 | 1.00 /1.00 | 0.00 / 0.02 | 0.51 |

Off-diagonal elements are estimates of R-squared / Lewontin´s D´(assuming Hardy-Weinberg equilibrium). Diagonal are relative frequencies of allele 2.

The distribution of homozygous and heterozygous carriers corresponded to the expectations from the Hardy-Weinberg equilibrium (*P*>0.25 for each SNP in the discovery cohort, comparable in replication cohorts).
