# Supplementary material for: Genetic Analyses Reveal a Role for Vitamin D Insufficiency in HCV-Associated Hepatocellular Carcinoma Development
Source: PLoS One. 2013 May 29;8(5):e64053. doi: 10.1371/journal.pone.0064053 (PMC3667029; doi:10.1371/journal.pone.0064053)
Supplement: Table S2 — Primers for SNP genotyping assays. (DOC) [file pone.0064053.s002.doc]

**Table S2. Primers for SNP genotyping assays.**

| **rs ID** | **Allele X Primer** | **Allele Y Primer** | **Reverse Primer** | **Allele X** | **Allele Y** |
| --- | --- | --- | --- | --- | --- |
| rs2282679 | CAAAGCTAACAATAAAAAATACCTGGCTG | CAAAGCTAACAATAAAAAATACCTGGCTT | CATGYCCAGCAAATCTCTGTCTCTTAA | G | T |
| rs10741657 | TGGGGAGATACTTTAGCAGGCA | GGGGAGATACTTTAGCAGGCG | GCTCCAATGTCATCTTCCTAATAAGACTT | A | G |
| rs12785878 | GTCTGATATCACAAAGCTTCG | GGCTGTCTGATATCACAAAGCTTCT | CCACGGGGCCAGGAGAGGAT | G | T |
| rs1993116 | ATTCATATTCTTGTCTTCTGCCTGCA | CATATTCTTGTCTTCTGCCTGCG | GAAATCACTGCTCCTAAGGAGTTTGTAAT | A | G |
