# Supplementary material for: Genetic Analyses Reveal a Role for Vitamin D Insufficiency in HCV-Associated Hepatocellular Carcinoma Development
Source: PLoS One. 2013 May 29;8(5):e64053. doi: 10.1371/journal.pone.0064053 (PMC3667029; doi:10.1371/journal.pone.0064053)
Supplement: Table S3 — Summary of associations between SNPs in CYP2R1 , GC , and DHCR7 , and HCV-related hepatocellular carcinoma development, considering the SCCS as case-control study. (DOC) [file pone.0064053.s003.doc]

**Table S3. Summary of associations between SNPs in *CYP2R1*, *GC*, and *DHCR7*, and HCV-related hepatocellular carcinoma development, considering the SCCS as case-control study.**

| ***CYP2R1*** |  |  | **Cases** | | | **Controls** | | | **Risk allele frequencies** | |  |  |
| --- | --- | --- | --- | --- | --- | --- | --- | --- | --- | --- | --- | --- |
| **SNP** | **Study** | **Allele 1/2** | **11** | **12** | **22** | **11** | **12** | **22** | **Case** | **Control** | ***P*** | **OR (95% CI)** |
| rs1993116 | SCCS | A/G | 8 | 29 | 35 | 246 | 920 | 756 | 0.69 | 0.63 | 0.11 | 1.46 (0.96-1.45) |
| rs1993116 | Japanese  GWAS | A/G | 41 | 136 | 133 | 163 | 621 | 468 | 0.65 | 0.62 | 0.07 | 1.26 (0.98-1.61) |
| rs10741657 | Japanese Replication | A/G | 106 | 377 | 320 | 174 | 597 | 482 | 0.63 | 0.62 | 0.5 | 1.06 (0.88-1.27) |
| rs1993116 | Bonn-Berlin | A/G | 17 | 48 | 47 | 25 | 98 | 81 | 0.63 | 0.64 | 0.7 | 1.10 (0.67-1.76) |
|  | ***Combined*** | A/G | ***172*** | ***590*** | ***535*** | ***608*** | ***2236*** | ***1787*** | ***0.64*** | ***0.63*** | ***0.08*** | ***1.12 (0.99-1.27)*** |
| ***GC*** |  |  |  |  |  |  |  |  |  |  |  |  |
| **SNP** | **Study** | **Allele 1/2** | **11** | **12** | **22** | **11** | **12** | **22** | **Case** | **Control** | ***P*** | **OR (95% CI)** |
| rs2282679 | SCCS | T/G | 34 | 32 | 6 | 1024 | 759 | 129 | 0.31 | 0.27 | 0.6 | 1.26 (0.56-2.7) |
| rs2282679 | Japanese  GWAS* | T/G | 153 | 125 | 31 | 679 | 475 | 97 | 0.30 | 0.27 | 0.19 | 1.33 (0.87-2.03) |
| rs2282679 | Bonn-Berlin | T/G | 55 | 46 | 15 | 117 | 77 | 14 | 0.33 | 0.25 | 0.06 | 2.01 (0.97-4.38) |
|  | ***Combined*** | ***T/G*** | ***242*** | ***203*** | ***52*** | ***1820*** | ***1311*** | ***240*** | ***0.31*** | ***0.27*** | ***0.008*** | ***1.56 (1.11-2.01)*** |
| ***DHCR7*** |  |  |  |  |  |  |  |  |  |  |  |  |
| **SNP** | **Study** | **Allele 1/2** | **11** | **12** | **22** | **11** | **12** | **22** | **Case** | **Control** | ***P*** | **OR (95% CI)** |
| rs7944926 | SCCS | T/C | 21 | 16 | 4 | 525 | 356 | 65 | 0.29 | 0.26 | 0.6 | 1.19 (0.63-2.22) |
| rs7944926 | Japanese  GWAS | T/C | 127 | 156 | 27 | 599 | 534 | 119 | 0.34 | 0.31 | 0.03 | 1.32 (1.03-1.70) |
| rs12785878 | Japanese Replication | T/G | 84 | 336 | 383 | 153 | 543 | 557 | 0.69 | 0.66 | 0.15 | 1.13 (0.95-1.36) |
| rs12785878^2^ | Bonn-Berlin | T/G | 63 | 44 | 9 | 113 | 77 | 18 | 0.27 | 0.27 | 1.00 | 1.00 (0.63-1.58) |
|  | ***Combined^#^*** | ***T/C*** | ***148*** | ***172*** | ***31*** | ***1124*** | ***890*** | ***184*** | ***0.33*** | ***0.29*** | ***0.002*** | ***1.44 (1.14-1.80)*** |

The analyses shown here are similar than the analyses in Table 2 of the main document with the difference, that the inclusion criterion “known duration of infection” was omitted for selecting SCCS patients. Hence, the SCCS in these analyses represents a case-control study like the three other cohorts as well.

Allele 2 indicates the risk allele, according to Wang et al. . *P*-values and ORs were calculated for risk genotypes using favorable genotypes as a reference, i.e. for *CYP2R1* by comparing GG *vs.* GA/AA genotypes, for *GC* by comparing TT/TG vs. GG genotypes, and for *DHCR7* by comparing TT vs. TC/CC genotypes. ^#^Only patients from the SCCS and Japanese GWAS were included in the combined analysis for this locus, because of the different allele frequencies for rs12785878 in Japanese patients compared to Caucasian patients. Data remain significant after inclusion of the Bonn-Berlin cohort (P=0.014, OR=1.28 [95% CI=1.05-1.57]). *Genotyping of this SNP failed in the Japanese Replication cohort due to limited amounts of DNA. Please note that the total number of patients with available genotypes is not equal between different loci due to limited amount of DNA or genotyping failure in some cases.
